# HDPE/Chitosan Blends Modified with Organobentonite Synthesized with Quaternary Ammonium Salt Impregnated Chitosan

**DOI:** 10.3390/ma11020291

**Published:** 2018-02-13

**Authors:** Maria José G. de Araújo, Rossemberg C. Barbosa, Marcus Vinícius L. Fook, Eduardo L. Canedo, Suédina M. L. Silva, Eliton S. Medeiros, Itamara F. Leite

**Affiliations:** 1 Graduate Program in Science and Materials Engineering, Federal University of Paraíba, João Pessoa, PB 58051-900, Brazil; maria.quimicaindustrial@yahoo.com.br; 2 Department of Materials Engineering, Federal University of Campina Grande, Campina Grande, PB 58429-900, Brazil; rcbvet@gmail.com (R.C.B.); marcus.fook@pq.cnpq.br (M.V.L.F.); ecanedo2004@yahoo.com (E.L.C.); suedina.silva@ufcg.edu.br (S.M.L.S.); 3 Department of Materials Engineering, Federal University of Paraíba, João Pessoa, PB 58051-900, Brazil; elitonsdemedeiros@gmail.com

**Keywords:** HDPE, chitosan, organobentonite, compatibilizer, quaternary ammonium

## Abstract

In this study, blends based on a high density polyethylene (HDPE) and chitosan (CS) were successfully prepared by melt processing, in a laboratory internal mixer. The CS biopolymer content effect (up to maximum of 40%), and, the addition of bentonite clay modified with quaternary ammonium salt (CTAB) impregnated chitosan as a compatibilizing agent, on the properties of the blends was analyzed by Fourier transform-infrared spectroscopy (FT-IR), wide angle X-ray diffraction (WAXD), differential scanning calorimetry (DSC), thermogravimetric analyses (TG), tensile strength, and scanning electron microscopy (SEM). The use of clay modified with CTAB impregnated chitosan, employing a method developed here, improved the compatibility of HDPE with chitosan, and therefore the thermal and some of the mechanical properties were enhanced, making HDPE/chitosan blends suitable candidates for food packaging. It was possible to obtain products of synthetic polymer, HDPE, with natural polymer, chitosan, using a method very used industrially, with acceptable and more friendly properties to the environment, when compared to conventional synthetic polymers. In addition, due to the possibility of impregnated chitosan with quaternary ammonium salt exhibit higher antibacterial activity than neat chitosan, the HDPE/chitosan/organobentonite blends may be potentially applied in food containers to favor the preservation of food for a longer time in comparison to conventional materials.

## 1. Introduction

Polyethylene (PE) is a common cheap polymer with gas and humidity barrier properties, good thermal stability, and excellent chemical resistance, and is used for short life products, like biaxially oriented films for packaging, blown molded bottles for food, carrier bags, food wrapping material, and so on [1,2,3]. However, PE is one of the primary components of municipal plastic waste and it is either non-biodegradable or decompose after long periods of time producing toxic derivatives, which can lead to severe problems to both human and animals [4,5,6,7,8,9]. In addition, PE packaging does not have antimicrobial and antifungal activities and bivalent minerals chelating ability [2,3,10,11,12]. 

The preparation of mixtures of non-bio-based polymer as PE and bio-based polymer as chitosan, represent a simple way to combine their best properties, in order to obtain materials with good morphological, thermal, and mechanical properties, and with a shorter degradation time when compared to non-bio-based polymers. This alternative could contribute to minimize environmental pollution, and, whereas chitosan has strong antimicrobial and antifungal activities, as reported by several researchers, the conduction of this study may enable the extension of PE applications for packaging material [13,14,15,16,17,18,19,20,21,22,23,24,25,26]. 

In view of the above mentioned considerations, many researchers are working on blends of chitosan with various polymers either for increasing the bio-based content or for the antimicrobial effect of chitosan. Thus, the development of research about blends of chitosan with polyolefins has received considerable attention, especially those that are prepared by melt processing, because of their high yield, and greater control of the final material’s characteristics without atmospheric pollution, in comparison with the solvent evaporation methods [12,13,14,27,28,29,30,31,32,33,34,35,36,37,38]. However, there are only a few published reports on blends of chitosan with high density polyethylene (HDPE) [38,39], and, to the best of our knowledge, the compatibilization of these blends with organobentonite synthesized with quaternary ammonium salt impregnated chitosan has not been conducted yet.

Although it is difficult to assess the kind and degree of interaction between chitosan and quaternary ammonium salts, such as cetyl trimethyl ammonium bromide (CTAB), which probably does not involve covalent bonding, a close association—which may be called “impregnation” has been suggested in the literature [40]. Thus, the incorporation of organobentonite synthesized with CTAB impregnated chitosan into the blend of HDPE/chitosan can result in good interfacial adhesion between chitosan and the polymer matrix. In addition, it is established in the literature that the combination of ammonium salts with chitosan retains its properties and improves its natural antibacterial and antifungal properties [41,42,43].

In light of this, blends of HDPE with chitosan have been prepared in this study by melt processing, using different formulations of the components. An organobentonite synthesized with CTAB impregnated chitosan, using two different methods—evaporation and precipitation, has been added to the blends. The aim is to improve the compatibility between HDPE and chitosan and to increase the bio-based content in non-bio-based polymer.

## 2. Results and Discussion

**Figure 1:**
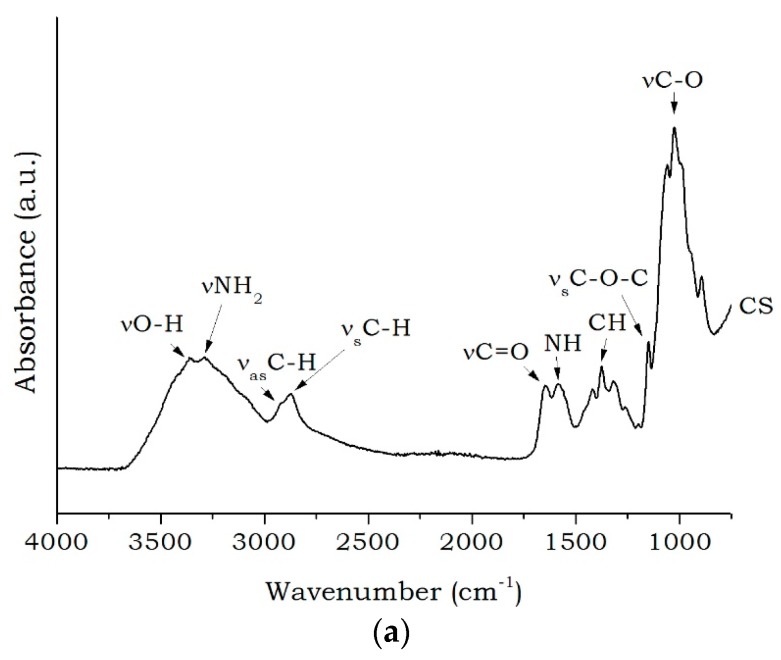

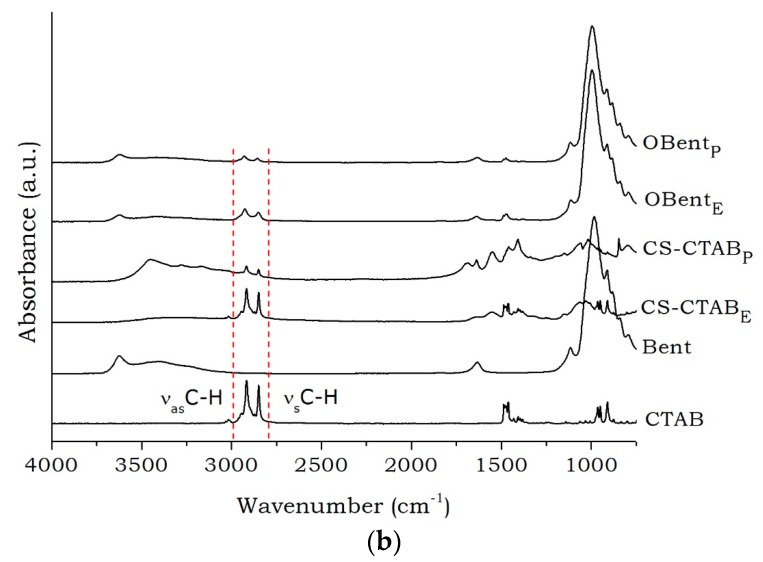
FTIR spectra of (**a**) pure CS and (**b**) CTAB salt, Bent clay, CS-CTAB_E_, CS-CTAB_P_, OBent_E_ and OBent_P_.

Figure 1 shows the spectra of pure chitosan (CS), organic salt (CTAB), natural bentonite clay (Bent), CTAB impregnated chitosan using two different methods—evaporation and precipitation (CS-CTAB_E_ and CS-CTAB_P_), and bentonite modified with CS-CTAB_E_ and CS-CTAB_P_ (OBent_E_ and OBent_P_).

In the pure chitosan (CS) spectrum, a broad band between 3000 and 3600 cm^−1^ was concerned as N-H and hydrogen bonded O-H stretch vibrations. Bands at 2915 and 2870 cm^−1^ were assigned to the asymmetrical and symmetrical C-H stretching, respectively. Strong bands at 1650 cm^−1^ and 1585 cm^−1^ could be assigned to the vibration of C=O bond and the deformation of the NH bond. C-H deformation was observed at 1419 cm^−1^ and the bands at 1365 cm^−1^ (C-N group) and 1314 cm^−1^ is related to the O-H and C-H bond present on the glycosidic ring [44]. The band at 1256 cm^−1^ was associated to the C-O bonds of ether [45]. The band at 1150 cm^−1^ corresponds to the symmetric stretching of C-O-C and the peaks from 1150 to 900 cm^−1^ were assigned to the amino group on the C2 position of pyranose ring [46,47]. Similarly to pure Chitosan (CS), two peaks around 2923 cm^−1^ and 2843 cm^−1^ appeared in the spectrum of ammonium trimethyl cetyl bromide salt (CTAB) (Figure 1b), which were assigned to the asymmetrical and symmetrical C-H stretching, respectively, and, at 1470 cm^−1^, an asymmetric deformation of the C-H group was also observed [48].

In the CTAB impregnated chitosan spectra, CS-CTAB_E_ and CA-CTAB_P_, (Figure 1b), regardless the method used, it was observed the presence of the bands around 2923 and 2843 cm^−1^, associated to the asymmetric and symmetric modes of the C-H group. Another band in the region of 1470 cm^−1^, which is characteristic of the asymmetric deformation of the C-H group belonging to the CTAB salt, was also observed. This indicates that CTAB molecules have interacted with both amino and hydroxyl groups of CS molecules during impregnation and are in accordance with the findings of Chatterjee et al. [40]. In comparative analysis, it can be verified that the bands referring to the asymmetric and symmetric stretching of the C-H group show differences in their intensities due to the production method. They were more intense for chitosan that was impregnated with CTAB by evaporation method (CS-CTAB_E_). Such behavior can be attributed to a higher content of CTAB salt that is present in modified chitosan prepared by this method. In addition, for CS-CTAB_P_ sample spectrum it is also observed the presence of a band around 3450 cm^−1^ associated with the OH group present in the chitosan, as well as in the NaOH solution used in the precipitation process.

After chemical modification of the bentonite clay with CTAB impregnated chitosan (OBent_E_ and OBent_P_), the presence of bands around 2930 and 2850 cm^−1^, related to the asymmetrical and symmetrical stretches of the C-H group, can be identified (Figure 1b). The differences in their intensities due to the production method was also observed. More intense bands were noted for OBent_E_ that were due, maybe, to the higher content of CTAB salt present in modified chitosan, as prepared by evaporation method. At 1600 cm^−1^, a band associated with the presence of adsorbed water in the clay was also found. However the bentonite clay modified with CTAB impregnated chitosan resulted in a slight decrease in the intensity of this band, evidencing a decrease in the hydrophilic character of the clay [49]. These results indicate that the evaporation method was more efficient to prepare the CTAB impregnated chitosan as bentonite clay modified both with chitosan and CTAB.

**Figure 2:**
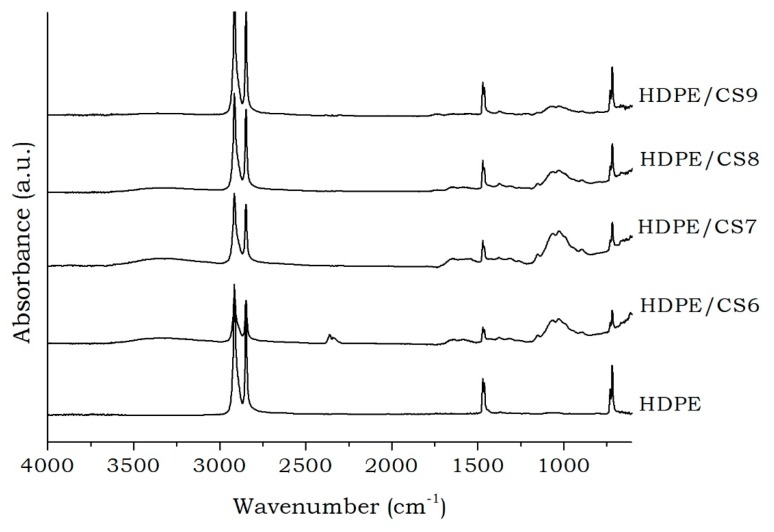
FTIR spectra of High density polyethylene (HDPE) and HDPE/chitosan (CS) formulations.

For all HDPE/CS formulations, it was possible to observe the presence of the absorption bands that were belonging to both HDPE and CS (Figure 2). Moreover, neither new chemical bonds were formed on the polymers mixture nor notable shifts of absorption bands were observed, suggesting that this mixture presented immiscible character [50]. It should be remembered that all spectra of the HDPE/CS mixtures, regardless the proportions and compatibilizing agents (OBent_E_ and OBent_P_) that were used, showed similar behavior, and for this reason it will not be presented here.

**Figure 3:**
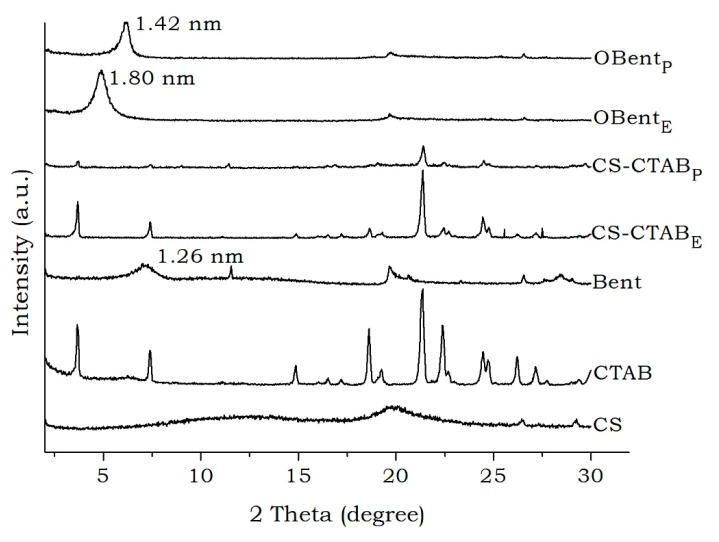
X-ray diffraction pattern of CS, ammonium trimethyl cetyl bromide (CTAB) salt, Bent clay, CS-CTAB_E_, CS-CTAB_P_, OBent_E_, and OBent_P_.

The XRD patterns of CS, CTAB salt, Bent clay, CS-CTAB_E_, CS-CTAB_P_, OBent_E_, and OBent_P_ are shown in Figure 3. Pure CS shows the characteristic crystalline peaks around 2*θ* = 9–13°, 20°, 26°, and 29°. The peaks around 10° and 20° are related to crystal (1) and crystal (2) in chitosan, respectively. The unit cell of crystal (1) is characterized by a = 7.76, b = 10.91, c = 10.30 Å, and *β* = 90°, and it is larger than that of crystal (2) whose unit cell is characterized by a = 4.40, b = 10.00, c = 10.30 Å, and *β* = 90° [51,52].

Bent natural clay showed several typical reflections at 2*θ* = 7°, 20°, 26°, and 28°, belonging to montmorillonite, which is present in higher quantity in this clay. Other peaks at 2*θ* = 12°, 21°, 27°, and 29° were also observed, typical of impurities as kaolinite and quartz [49].

As shown in Figure 3, the crystalline peaks belonging to the CTAB salt are also present in both samples, CS-CTAB_E_ and CS-CTAB_P_, suggesting the effective chitosan chemical modification, especially for the modified chitosan by evaporation method, where higher intensity crystalline peaks, which are characteristic of the CTAB salt, were evidenced. The low peaks intensity presented by modified chitosan by precipitation method can be attributed to the smaller amount of CTAB salt that is present in this sample. 

After modification of the bentonite with CTAB impregnated chitosan, it was observed that the 2*θ* angle for OBent_E_ and OBent_P_ was shifted to smaller angles, from 7° (Bent) to 4.89° (OBent_E_) and 6.22° (OBent_P_), increasing the d(001) basal spacing of Bent clay from 1.26 nm to 1.80 nm (OBent_E_) and 1.42 nm (OBent_P_). This suggests that the chemically modified Bent clay with CS-CTAB_E_ and CS-CTAB_P_, regardless the method employed, was incorporated into the lamellae of clay [53,54]. Considerable increase in basal spacing, around 43%, was observed, especially for chemically modified clay with CS-CTAB_E_. Such behavior can be attributed to higher content of CTAB salt that is present in modified chitosan prepared by precipitation method, as suggested by FTIR data. Figure 3 also shows a reduction in the intensity of the basal reflections at 2*θ* = 12°, 21°, 27°, and 29°, which is characteristic of impurities, indicating that the washing during the chemical modification process of the clays may have been efficient in decreasing the amounts of impurities present in them.

The intercalation of organic species into layered inorganic solids provides an useful and convenient route to prepare organic-inorganic hybrids that contain properties of both the inorganic host and the organic guest in a single material [55]. In the last years, smectite clays that are intercalated by molecules have attracted great interest from researchers since they show new physical and chemical properties [56]. In the present work, chitosan and ammonium quaternary salt were successfully intercalated into layered silicate that was used as compatibilizing agent to prepare mixtures of HDPE/CS. 

**Figure 4:**
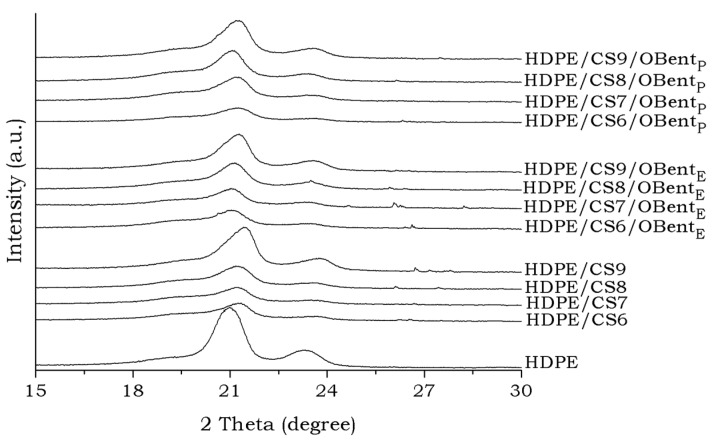
X-ray diffraction pattern of HDPE, HDPE/CS, and HDPE/CS/Obent formulations.

Figure 4 shows the XRD pattern for HDPE and all HDPE/CS formulations. For HDPE, it is observed the presence of two crystalline peaks at 21° and 23.3°, related to the (110) and (200) planes, respectively, characteristics of the orthorhombic structure of the crystal polyethylene [57,58]. For all the HDPE/CS formulations, with and without compatibilizing agent (OBent_E_ and OBent_P_), crystalline peaks were observed in the region of 21° and 23°, which is characteristic of the crystalline phase of the HDPE and another peak of low intensity around 20° belonging to chitosan, as expected. By comparing all the XRD patterns, a slight decrease in the intensity of the crystalline peaks of the HDPE/CS mixtures was observed as the chitosan content is increased. This behavior may be associated with the chitosan structure to clutter the packaging of the HDPE molecules, thus reducing the crystallinity index [58].

According to Table 1, the degree of crystallinity (Xc) for all the HDPE/CS formulations is in general lower than that presented by HDPE, since the addition of chitosan in the HDPE affects the crystalline arrangement of the polymer molecules, corroborating with the discussion that is described above. Similar behavior has also been observed by Carrasco-Guigón et al. [24] using PP/Chitosan composites.

Table 2 shows the thermal properties determined by differential scanning calorimetry (DSC) under heating and cooling for HDPE and HDPE/CS formulations. It is observed that the addition of different contents of chitosan and compatibilizing agents (OBent_E_ and OBent_P_) in the HDPE matrix did not have any important effect on the Tm and Tc. Similar behavior has been reported in the literature.

The crystallinity of HDPE decreased with the presence of different chitosan and compatibilizing agents (OBent_E_ and OBent_P_) contents in the HDPE matrix, as observed by XRD data, which indicates that the chitosan particles settled between the HDPE chains, hindering the ordering of the HDPE chains [34,59]. According to Mir et al., the crystallinity is greatly affected by the incorporation of chitosan. It shows that chitosan inhibits the close packing of the HDPE chains.

In this study, thermogravimetric analysis (TG) was conducted to assess the effect of chitosan (CS) and bentonite modified with chitosan (OBent_E_ and OBent_P_) addition on the thermal properties of HDPE. Thermal stability studies are necessary in the case of materials that are used for packaging application as the polymer may be subjected to heat processes during their preparation, processing, or conception. Table 3 shows the temperatures of the degradation step of the HDPE, CS, and HDPE/CS, with and without compatibilizing agent (OBent_E_ and OBent_P_). 

The HDPE showed a single degradation step within a temperature range of 463–489 °C, whereas CS and the blends HDPE/CS and HDPE/CS/OBent showed three degradation steps (Table 3). The first step below 100 °C was mainly attributed to the loss of absorbed water from the materials. The second step, ranging from 260 °C to 314 °C, was due to thermal degradation of chitosan. In this stage, degradation of chitosan took place, which involved dehydration, deacetylation, and chain scission reactions. The third step (406–490 °C) was attributed to the decomposition of the HDPE matrix. The data are in accordance with those that were found by other authors [60]. 

It is clear from the Table 3 data that the thermal stability of HDPE/CS and HDPE/CS/OBent blends are like that of HDPE. Chitosan and chitosan-organomodified bentonite additives had no significant effect on the overall characteristics of each weight loss of the HDPE-based materials, regardless the method (evaporation or precipitation) or the weight ratio used. 

Since the degradation of HDPE was not affected when blended with chitosan and chitosan-organomodified bentonite, the blends HDPE/CS and HDPE/CS/OBent prepared by melt processing may perhaps avoid degradation and/or a drastic loss in the antibacterial properties of the final material prepared by melt processing [61].

The variations of the tensile strength, Young’s modulus and elongation at break properties of HDPE, HDPE/CS, and HDPE/CS/OBent formulations are presented in Table 4. In comparative analysis, it is verified that the tensile strength of HDPE was not affected when blended with chitosan and chitosan-organomodified bentonite, regardless the additive type and its quantity. Still, incorporation of CS and OBent in the HDPE matrix led to an increase of Young’s modulus in respect with that of the neat HDPE, and its value increases for higher chitosan amount, but it was not affected by OBent preparation method (evaporation or precipitation). This behavior can be explained based on the rigidity of chitosan. Other papers reported in the literature [34] based on PE and chitosan blends, showed that the mechanical properties decreased except for the Young’s modulus, which increased with the addition of chitosan because this biopolymer is more rigid than PE. The samples HDPE/CS, for all studied chitosan amount, are strong materials with low ductility, when compared with neat HDPE, but they do not behave as brittle samples. For HDPE/CS/OBent samples that are prepared with until 20% (*w*/*w*) of OBent, particularly OBent_P_, the higher elongation at break values were obtained (Table 4).

**Figure 5:**
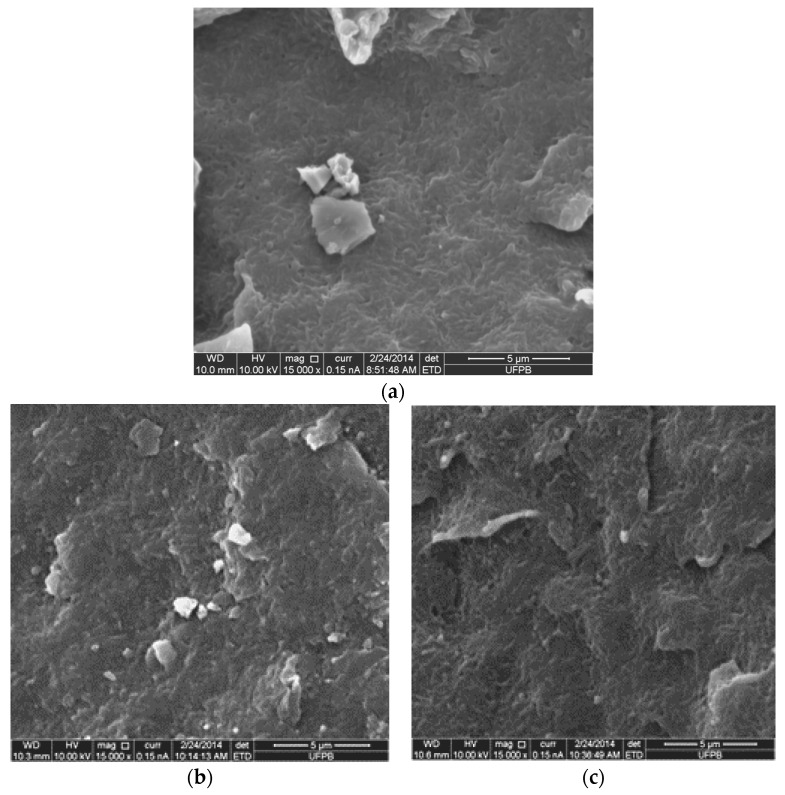
Scanning electron micrograph of (**a**) HDPE/CS8; (**b**) HDPE/CS8/OBent_E_; and (**c**) HDPE/CS8/OBent_P_.

In view of the results obtained in the tensile tests and aiming to investigate the interactions between HDPE and chitosan, scanning electron microscopy (SEM) analyzes were performed and the micrographs are shown in Figure 5.

When compared to HDPE/CS8, for the samples HDPE/CS8/OBent_E_, and, particularly for HDPE/CS8/OBent_P_, we can see a reduction in chitosan particle size and their better dispersion in the HDPE matrix, indicating a good homogeneity and interfacial adhesion between HDPE and chitosan. These results corroborate with the mechanical properties data presented in Table 4. In view of this, it is suggested that bentonite clay modified with cetyl trimethyl ammonium bromide that is impregnated chitosan played a significant role as compatibilizer in the decreasing the domain sizes of the dispersed phase and reducing the interfacial tension. Thus, contributing to a better interfacial adhesion between HDPE and chitosan used in this study.

## 3. Materials and Methods

### 3.1. Materials

High density polyethylene (HDPE), GM9450F, with melt flow index of 9.3 g/10 min (190 °C/2.16 kg) was purchased from Braskem, São Paulo, Brazil [62]. Chitosan (CS) from crab shells, as supplied by Polymar, Leola, PA, USA, with 95% deacetylation degree was used since the high amount of active primary amine on the chitosan backbone provide excellent reactive sites for chemical modifications. Moreover, highly deacetylated chitosan is more antimicrobial than acetylated chitosan, as reported in the literature [63]. Argel natural bentonite (Bent), with cation exchange capacity of 0.92 meq/g, as determined via methylene blue test according to ANSI/ASTM C837-99 [64], was obtained from Bentonit União Nordeste, Campina Grande, Brazil. This bentonite was passed through a 200 mesh sieve and was used without further purification. XRD patterns showed that it was mainly composed of sodium montmorillonite (>95%) [65]. The quaternary ammonium salt used was cetyl trimethyl ammonium bromide (C_16_H_33_(CH_3_)_3_N^+^Br^−^, FW: 364 g/mol), labelled CTAB, supplied by Vetec, Remscheid, Germany, and it was used without any further purification. Analytical grade reagents (glacial acetic acid and sodium hydroxide) were purchased from Vetec and were used as received. All of the solutions were prepared with distilled water throughout the experiment. 

### 3.2. Preparation of CTAB Impregnated Chitosan

The CTAB impregnated chitosan (CS-CTAB) was prepared as follows: chitosan solution was made by dissolving the required amount of chitosan powder (3 g) into 1% aqueous acetic acid solution (300 mL). The reactants were magnetically stirred at 45 °C for 2 h, and then the CTAB (0.0662 g) was added to the reaction mixture, and stirred at the same temperature for another 2 h. The CTAB amount was determined to react with 100% of the amino groups present in the chitosan and was calculated as:

(1) $$\phi =\frac{\alpha M_{S}}{M_{E}+\alpha M_{1}+(1-\alpha )M_{2}}$$

here *φ* is the amount of CTAB necessary to react with 1 g of chitosan; *M_S_*, *M_E_*, *M*_1_, and *M*_2_ are the molar mass of CTAB, glucose skeleton, amino group, and acetamide group, respectively, and *α* = 0.95 is the deacetylation degree.

When the reaction was complete, two different methods for obtaining CS-CTAB powders were employed: evaporation and precipitation. For the first method, the CS-CTAB reaction mixture was oven dried under air circulation at 50 °C for 115 h, subsequently grounded with an agate mortar and sieved to 74 μm for use. For the precipitation method, the CS-CTAB reaction mixture was maintained at room temperature (24 h), then it was precipitated via the addition of NaOH solution 1 M (130 mL), followed by centrifugation at 3800 rpm for 5 min at room temperature. The precipitated material was collected, oven dried under air circulation at 50 °C for 96 h, and also grounded and sieved in the conditions previously described. The CS-CTAB powders that were obtained by evaporation and precipitation were coded as CS-CTAB_E_ and CS-CTAB_P_, respectively.

### 3.3. Preparation of CS-CTAB Modified Bentonite

Modified bentonite (organobentonite) was synthesized by an ion exchange reaction, adding 7.0 g of CS-CTAB_E_ and CS-CTAB_P_ into a dispersion of 2.0 g of bentonite in 100 mL water. The mixture was stirred at 70 °C for 30 min, and then cooled down to room temperature and maintained at this temperature for 24 h. Next, the samples were vacuum filtered and washed with distilled water (2 L). The filter cake was dried at 60 °C for 48 h, grounded and sieved to 74 μm for use. The organobentonites prepared with CS-CTAB_E_ and CS-CTAB_P_ were coded as OBent_E_ and OBent_P_, respectively.

The mass of CTAB impregnated chitosan was computed by:

(2) $$CS-CTAB(g)=\frac{MM_{CS-CTAB(meq)}}{1000}$$

where *MM_CS-CTAB_*_(*meq*)_ was determined according to:

(3) $$MM_{CS-CTAB(meq)}=\frac{MM_{CS-CTAB}}{h}$$

where *MM_CS-CTAB_* is the molar mass of *CS-CTAB* and *h* is the valence of the cation.

### 3.4. Preparation of HDPE Compounds

Melt processing of neat HDPE and the corresponding compounds with chitosan (CS), CTAB functionalized chitosan (CS-CTAB), and CS-CTAB modified bentonite (OBent) has been realized by means of a Haake Rheomix 3000 laboratory internal mixer, equipped with a mixing chamber with high intensity rotors (roller type) operated at 60 rpm with the chamber wall maintained at 170 °C. Batch mass was selected to fill 90% of the processing chamber volume during the last stage of the process (fully molten material). HDPE, as received, was processing for 10 min. For compounds, HDPE was loaded first and after 6 min, CS, CS-CTAB, and OBent (dried under vacuum for 48 h at 50 °C) were added without interrupting the process and the mixing continued for another 4 min. After the melt processing, each sample was grounded, dried under vacuum for 24 h at 80 °C, and pressed between two Teflon sheets in a hot-plate hydraulic press, for 5 min at 160 °C under 12 ton, to obtain the specimens in the form of plates for the mechanical, FTIR, and WAXD tests. The prepared samples formulations are summarized in Table 5.

### 3.5. Characterization

Fourier transform infrared with attenuated total reflection (FTIR-ATR) spectra were used to characterize the pristine materials (CS, Bent, CTAB, and HDPE), modified materials (CS-CTAB and OBent) and the compounds (HDPE/CS, HDPE/CS-CTAB, and HDPE/OBent) by means of the 8400S Shimadzu instrument (Tokyo/Kyoto, Japan). All spectra were an average of 64 scans at a resolution of 4 cm^−1^ from 400 cm^−1^ to 4000 cm^−1^, and determined at 25 °C using the Opus software. Chitosan, bentonite, and cetyl trimethyl ammonium bromide samples were characterized as powder sieved through a 200 mesh, and pure HDPE and HDPE compounds were characterized in the form of plates.

Wide angle X-ray diffraction (WAXD) was used to estimate the basal spacing (*d*_001_) of bentonite and HDPE crystallinity. The measurements were performed, at room temperature, on a Siemens D5000 X-ray diffractometer (Houston, TX, USA) with CuK*α* radiation (*λ* = 1.54056 Å). The generator was operated at 40 kV and 40 mA. The spectra were recorded over a 2*θ* range of 2°–10° and 15°–30° for bentonites and HDPE, respectively, using a scan rate of 0.02°/s. The basal spacing (*d*_001_) value of bentonite was calculated through Bragg’s law [49]:

(4) $$d_{001}=\frac{\lambda }{2\sin\theta }$$

where *θ* is the measured diffraction angle.

The HDPE degree of crystallinity was calculated from the WAXD data by following the Equation (5), using simple peak area method [66]:

(5) $$X_{c}(\%)=\frac{\mathrm{Intensity}\;\mathrm{of}\;\mathrm{crystalline}\;\mathrm{peaks}(s)}{\mathrm{Total}\;\mathrm{diffraction}\;\mathrm{intensity}}\times 100=\frac{I_{c}}{I_{c}+I_{a}}\times 100$$

where *I_c_* is the diffraction intensity associated with crystalline component and *I_a_* is the diffraction intensity that is associated to amorphous component.

Chitosan, bentonite, and cetyl trimethyl ammonium bromide samples were characterized by WAXD as powder sieved through a 200 mesh, and pure HDPE and HDPE compounds were characterized in the form of plates.

The crystallization and melting behavior of the samples was measured using a Shimadzu DSC (Kyoto, Japan)—60 calorimeter, under a continuous argon flow of 50 mL/min. The instrument was calibrated using an indium standard (Tm = 156.4 °C and Δ*H* = 28.4 J/g). The samples used for crystallization and melting study were pressed plates. The samples 5 mg were first heated from 30 °C to 200 °C, kept at 200 °C for 3 min to remove the effect of previous thermal, mechanical, processing, crystallization, and shear history. The melt was then cooled down to −130 °C at the rate of 10 °C min^−1^, and the resulting cooling curves or exotherms were recorded. Again, the samples were heated from 30 °C to 200 °C at the same heating rate to record the second melting curves. The second heating scans are used to determine the crystalline melting enthalpy of the matrix. The exothermic and endothermic curves were used to study the crystallization and melting characteristics of the HDPE matrix, respectively.

The heat of fusion was used to evaluate the degree of crystallinity (%) following Equation (6) for normalized sample of 5 mg while considering the enthalpy for 100% crystalline *HDPE*, (Δ*H_theoretical_* = 277 J/g) [67]:

(6) $$X_{c}(\%)=\frac{\Delta H_{experimental}/W_{HDPE}}{\Delta H_{theoretical}}\times 100$$

where *X_c_* is degree of crystallinity and *W_HDPE_* is weight fraction of HDPE in the composites.

The thermal stability of the samples was characterized under argon environment, using a Shimadzu 60H thermogravimetric analyzer (TG) (Kyoto, Japan). Samples of approximately 10 mg in open alumina crucibles were heated from 30 °C to 900 °C at 10 °C/min in a flow of 50 mL/min of argon gas; sample mass and temperature were recorded as function of time. 

Tensile tests were performed according to the ASTM D638 test methods. The properties were measured on a Shimadzu Universal Tester (Kyoto, Japan), Model autograph AG-X 10 KN, at a cross-head speed 5 mm/min. Average value of six samples was reported for each composition. All of the measurements were carried out at room temperature (30 ± 2 °C).

To evaluate the effects of adding organobentonite on the morphology of the HDPE/CS compounds, scanning electron microscopy (SEM) imagens were captured in a FEI Quanta 450 instrument (Hillsboro, OR, USA) on samples fractured in liquid nitrogen. A fine layer of gold was deposited over the fracture surfaces prior to test.

## 4. Conclusions

HDPE/chitosan blends with a maximum chitosan content of 40% (*w*/*w*) were prepared by melt mixing and organobentonite synthesized with quaternary ammonium salt impregnated chitosan, by precipitation and evaporation methods, was used as a compatibilizing agent. As shown by the FTIR and XRD data, the chitosan was successfully modified with ammonium trimethyl cetyl bromide salt (CTAB), and between the methods that were employed in the chitosan modification, the evaporation method was more efficient. The addition of the CTAB impregnated chitosan as bentonite clay modifier had a significant effect on the clay basal spacing increase, especially for chemically modified clay with CTAB functionalized chitosan by evaporation method, indicating that chitosan and ammonium quaternary salt were successfully intercalated into clay layers. The organobentonite clay improved both the compatibility of HDPE with chitosan, and as a consequence, thermal and some of mechanical properties were enhanced, making HDPE/chitosan blends suitable candidates for food packaging. It was possible to obtain products of synthetic polymer, HDPE, with natural polymer, chitosan, using a method very used industrially, with acceptable properties and more friendly to the environment when compared to conventional synthetic polymers. In addition, due to the possibility of modified chitosan with quaternary ammonium salt exhibit higher antibacterial activity than neat chitosan, the HDPE/chitosan/organobentonite blends may be potentially applied in food containers to favor the preservation of food for a longer time in comparison to conventional materials. Studies aiming at the elucidation of the antibacterial activity of these blends were initiated, and the results will be presented in the next publication.

## Figures and Tables

**Table 1:** Degree of crystallinity (Xc) of HDPE and HDPE/CS formulations, as calculated by XRD.

Sample	Xc (%)
HDPE	54
HDPE/CS9	45
HDPE/CS8	43
HDPE/CS7	38
HDPE/CS6	39
HDPE/CS9/OBent_E_	46
HDPE/CS8/OBent_E_	44
HDPE/CS7/OBent_E_	35
HDPE/CS6/OBent_E_	37
HDPE/CS9/OBent_P_	48
HDPE/CS8/OBent_P_	44
HDPE/CS7/OBent_P_	44
HDPE/CS6/OBent_P_	42

**Table 2:** Differential scanning calorimetry (DSC) data for high density polyethylene (HDPE), and HDPE/CS formulations.

Sample	Tc (°C)	∆Hc (J/g)	Tm (°C)	∆Hm (J/g)	Xc (%)
HDPE	115	155	130	184	64
HDPE/CS9	115	110	130	141	53
HDPE/CS8	114	40	129	99	42
HDPE/CS7	113	45	128	56	27
HDPE/CS6	114	50	127	63	36
HDPE/CS9/OBent_E_	114	78	130	104	39
HDPE/CS8/OBent_E_	115	65	129	90	38
HDPE/CS7/OBent_E_	114	57	129	68	33
HDPE/CS6/OBent_E_	115	51	129	64	36
HDPE/CS9/OBent_P_	115	91	129	106	40
HDPE/CS8/OBent_P_	115	79	129	95	41
HDPE/CS7/OBent_P_	113	60	127	71	35
HDPE/CS6/OBent_P_	114	47	128	68	39

**Table 3:** Characteristics temperatures of the degradation step of the HDPE, chitosan (CS), HDPE/CS, and HDPE/CS/OBent with different formulations.

Sample	T_i_–T_f_ (°C)		
	I	II	III
HDPE			463–489
CS	62–98	263–309	496–592
HDPE/CS9	40–78	263–311	459–490
HDPE/CS8	38–77	268–312	458–489
HDPE/CS7	36–78	265–310	451–486
HDPE/CS6	50–93	267–309	406–488
HDPE/CS9/OBent_E_	37–71	270–307	450–486
HDPE/CS8/OBent_E_	37–68	260–308	450–486
HDPE/CS7/OBent_E_	35–77	269–311	452–486
HDPE/CS6/OBent_E_	42–86	269–311	454–486
HDPE/CS9/OBent_P_	39–68	268–312	460–490
HDPE/CS8/OBent_P_	37–73	267–314	458–489
HDPE/CS7/OBent_P_	38–77	266–310	451–485
HDPE/CS6/OBent_P_	37–79	265–311	452–485

T_i_—onset temperature and; T_f_—end temperature.

**Table 4:** Tensile properties of HDPE, HDPE/CS, and HDPE/CS/OBent formulations.

Sample	Tensile Strength (MPa)	Young’s Modulus (MPa)	Elongation at Break (%)
HDPE	16.38 ± 2.11	400.72 ± 52.49	67.14 ± 12.33
HDPE/CS9	16.70 ± 0.58	443.50 ± 25.59	15.99 ± 0.65
HDPE/CS8	18.35 ± 0.98	538.28 ± 21.47	12.01 ± 3.92
HDPE/CS7	17.26 ± 0.45	537.47 ± 16.41	11.88 ± 0.88
HDPE/CS6	17.52 ± 0.60	597.84 ± 22.78	9.17 ± 1.35
HDPE/CS9/OBent_E_	17.19 ± 0.66	467.53 ± 13.09	20.15 ± 5.66
HDPE/CS8/OBent_E_	16.94 ± 2.95	531.29 ± 64.01	8.04 ± 2.13
HDPE/CS7/OBent_E_	17.99 ± 1.22	612.76 ± 43.71	7.10 ± 0.93
HDPE/CS6/OBent_E_	15.41 ± 1.33	565.76 ± 40.92	5.44 ± 0.29
HDPE/CS9/OBent_P_	15.38 ± 0.36	408.43 ± 11.41	20.57 ± 5.14
HDPE/CS8/OBent_P_	18.98 ± 0.65	554.68 ± 15.02	15.00 ± 3.38
HDPE/CS7/OBent_P_	15.74 ± 0.45	546.68 ± 42.41	7.58 ± 0.89
HDPE/CS6/OBent_P_	15.01 ± 1.05	567.82 ± 39.13	5.96 ± 0.32

**Table 5:** Formulation of the prepared samples.

Code	Composition	HDPE (g)	CS (g)	OBent_E_ * (g)	OBent_P_ * (g)
HDPE	100	50	0	0	0
HDPE/CS9	90/10	45	5	0	0
HDPE/CS8	80/20	40	10	0	0
HDPE/CS7	70/30	35	15	0	0
HDPE/CS6	60/40	30	20	0	0
HDPE/CS9/OBent_E_	90/10/0.1	45	5	0.05	0
HDPE/CS8/OBent_E_	80/20/0.2	40	10	0.10	0
HDPE/CS7/OBent_E_	70/30/0.3	35	15	0.15	0
HDPE/CS6/OBent_E_	60/40/0.4	30	20	0.20	0
HDPE/CS9/OBent_P_	90/10/0.1	45	5	0	0.05
HDPE/CS8/OBent_P_	80/20/0.2	40	10	0	0.10
HDPE/CS7/OBent_P_	70/30/0.3	35	15	0	0.15
HDPE/CS6/OBent_P_	60/40/0.4	30	20	0	0.20

* The content of OBent was 1% as related to CS mass.
